# TGFβ-induced changes in membrane curvature influence Ras oncoprotein membrane localization

**DOI:** 10.1038/s41598-022-17482-8

**Published:** 2022-08-05

**Authors:** Alexandros Damalas, Ivana Vonkova, Marijonas Tutkus, Dimitrios Stamou

**Affiliations:** 1 Department of Chemistry, University of Copenhagen, Copenhagen, Denmark; 2 Present Address: Fourth Department of Internal Medicine, School of Medicine, National and Kapodistrian University of Athens, Attikon University Hospital, 1st Rimini St, 12462 Haidari, Athens, Greece; 3 Present Address: CZ-OPENSCREEN, Institute of Molecular Genetics of the Czech Academy of Sciences, Vídeňská 1083, 142 20 Prague, Czech Republic; 4 Present Address: Institute of Biotechnology, Vilnius University, LT-10257 Vilnius, Lithuania; 5 Department of Molecular Compounds Physics, Center for Physical Sciences and Technology, LT-02300 Vilnius, Lithuania

**Keywords:** Oncogenes, Cancer, Membrane curvature, Small GTPases, Growth factor signalling

## Abstract

In the course of cancer progression tumor cells undergo morphological changes that lead to increased motility and invasiveness thus promoting formation of metastases. This process called epithelial to mesenchymal transition (EMT) is triggered by transforming growth factor (TGFβ) but for gaining the full invasive potential an interplay between signaling of TGFβ and Ras GTPases is required. Ras proteins possess a lipidated domain that mediates Ras association with the plasma membrane, which is essential for Ras biological functions. Type and number of the lipid anchors are the main difference among three Ras variants—H-ras, N-ras and K-ras. The lipid anchors determine membrane partitioning of lipidated proteins into membrane areas of specific physico-chemical properties and curvature. In this study, we investigated the effect of TGFβ treatment on the subcellular localization of H-ras and K-ras. We show that TGFβ increases positive plasma membrane curvature, which is subsequently sensed by H-ras, leading to its elevated plasma membrane localization and activation. This observation suggests the existence of a novel positive feedback loop whereby the increased level of plasma membrane curvature during TGFβ induced EMT attracts more Ras molecules to the plasma membrane resulting in increased Ras activity which in turn promotes further EMT and thus ultimately enables the acquisition of full invasive potential.

## Introduction

Ras proteins belong into superfamily of small GTPases. They function as tightly regulated GDP/GTP binary switches that control intracellular signaling networks: cytoskeleton integrity, cell proliferation, cell differentiation, cell adhesion, apoptosis, and cell migration. There are three distinct variants of Ras expressed in mammalian cells—H-ras, N-ras and K-ras. These three proteins share high degree of similarity but vary in differently post-translationally lipidated hypervariable domain in C-terminus. This domain is essential for Ras association with plasma membrane (PM) and therefore its biological function.

Upstream regulators of Ras proteins are several receptor tyrosine kinases such as EGFR, VEGFR or FGFR. After binding of their appropriate ligand, these receptors are autophosphorylated and recruit various proteins that serve as guanine nucleotide exchange factors (GEFs) or GTPase activated proteins (GAPs) for Ras. The recruitment of GEFs and GAPs to PM in direct proximity to Ras is essential for the tight regulation of Ras transition between active (GTP-bound) and inactive (GDP-bound) state^1^. Ras proteins are in a center of attention in cancer research for decades. Mutations in Ras GTP-binding domain that lock Ras in constitutively active state were among the first mutations associated with human cancer initiation and progression^2^. Moreover, Ras proteins represent the most frequently mutated oncogene family in human cancers. Mutations in at least one of the three isoforms were found in 9–30% of cancers^3,4^.

During cancer progression the cell morphology changes via transforming growth factor (TGFβ) induced process named epithelial to mesenchymal transition (EMT). EMT changes shape of cells from epithelial to mesenchymal which leads to increased motility and invasiveness of tumour cells and thus facilitates formation of metastases^5^. These alterations happen also in nanoscale as shown by decrease in the number of cellular protrusions observed by atomic force microscopy^6^.

The canonical TGFβ signaling pathway involves Smad transcription factors, however, TGFβ triggers also several non-Smad signaling pathways which include among others Ras proteins^7^. In the course of EMT Ras oncoprotein is activated and cooperative signaling between Ras and TGFβ, during which Ras plays prominent role in the switch from tumor-suppressive to tumor-promoting signaling of TGFβ, is important for maintenance of complete EMT and for gaining the full invasive potential of cancerous cells^8–11^.

Knowing the critical importance of Ras membrane localization for its biological functions, a precise knowledge of mechanisms that influence interaction of Ras with PM is key to understand Ras action and its regulation. It has been previously demonstrated that membrane partitioning of lipidated proteins, including N-ras, is sensitive to membrane curvature in vitro^12,13^. More recently evidences that Ras proteins directly sense membrane curvature also in vivo were provided^14^.

In this study we decided to investigate the effect of TGFβ treatment on subcellular localization of H-ras and K-ras. We concentrated on these two isoforms because of several reasons. Out of the three Ras isoforms H-ras was the first activated Ras gene detected and characterized and thus used in most of the studies, while K-ras is the most abundant and the most frequently mutated isoform^2–4,15,16^. In addition, both H-ras and K-ras form spatially non-overlapping nanoclusters and they show different sensitivity to membrane curvature with H-ras favoring more curved membranes than K-ras^14,17,18^. These findings raise the possibility that subcellular localization of H-ras and K-ras in response to TGFβ treatment may differ.

Here we present evidence that TGFβ treatment leads to elevated PM localization of H-ras and K-ras. Further investigation revealed that TGFβ induces increase in positive membrane curvature which is subsequently sensed by activated H-ras. Given the importance of interplay between Ras- and TGFβ-triggered signaling in cancer progression our findings suggest a possible mechanism how these two pathways can influence each other via triggering and subsequent sensing of changes in membrane curvature. It is important to highlight that the pathological significance of the observed effect of TGFβ-1 treatment on PM localization of different Ras isoforms in cancer progression remains yet to be explored.

## Results

### TGFβ-1 induces increase in Ras protein PM localization

**Figure 1:**
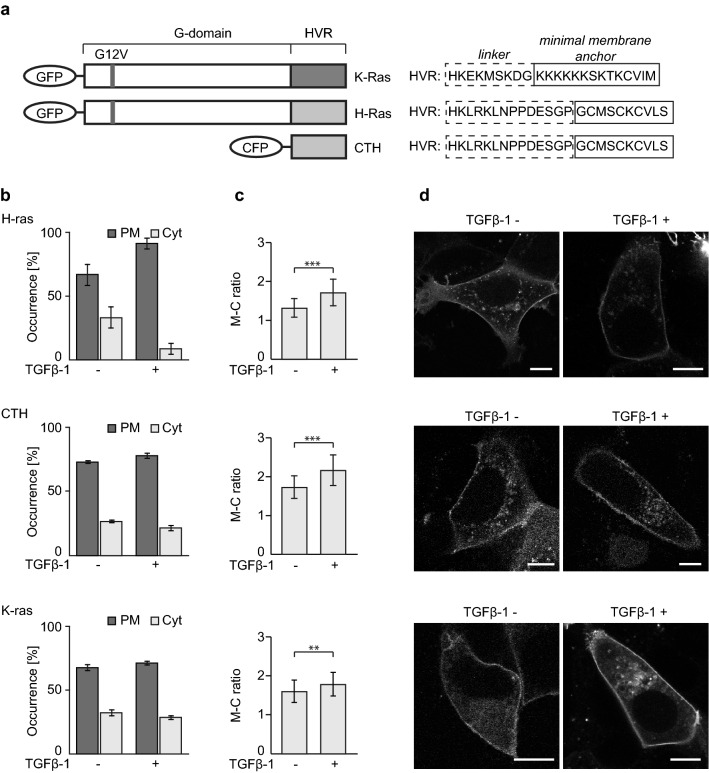
H-ras, CTH, and K-ras redistribute from the cytoplasm to the plasma membrane after TGFβ-1 treatment. Comparison of MCF7 cells expressing GFP-H-ras G12V (H-ras), CFP-CTH (CTH), or GFP-K-ras G12V (K-ras) treated or not with TGFβ-1 for 2 days. Data shown come from merging three independent biological replicates of each protein (for separate analysis of all replicates see Fig. S2). (**a**) Schematic representation of all Ras constructs used. HVR, hypervariable region. (**b**) Comparison of ratios between cells with cytoplasmic (Cyt) and plasma membrane (PM) localization (H-ras *n* ≥ 63, CTH *n* ≥ 62, K-ras *n* ≥ 159), error bars represent ± 0.5 SD. (**c**) Comparison of membrane-to-cytoplasm ratios (M-C ratio) (H-ras *n* ≥ 94, CTH *n* ≥ 80, K-ras *n* ≥ 199), error bars represent ± 0.5 SD, stars indicate statistically significant differences (*p = 0.01–0.05, **p = 0.001–0.009, ***p < 0.001; for exact *p*-values see Table S1). (**d**) Representative images of all tested conditions. Scale bar 10 μm.

To follow subcellular localization of H-ras and K-ras during TGFβ treatment we used breast cancer cell line MCF7 and transfected it with GFP tagged constitutively active GTP-bound H-ras G12V and K-ras G12V (Fig. 1a). The transfected cells were treated with TGFβ-1 for 2 days. The equal level of expression of fusion protein in samples with and without TGFβ-1 treatment was carefully checked and confirmed (Fig. S1a). The subcellular localization of fusion proteins was analyzed both qualitatively by visual inspection and quantitatively by calculating membrane-to-cytoplasm ratio (M-C ratio), when higher M-C ratio translated into higher accumulation of fusion protein on PM.

In case of H-ras a large proportion of cells showed cytoplasm localization in steady state and this proportion significantly decreased after TGFβ-1 treatment resulting in more than 91% of cells with H-ras on PM (Fig. 1b). In contrast, K-ras that showed a very similar proportion of cells with PM and cytoplasm localization in steady state as H-ras did not change this proportion even after TGFβ-1 addition. However, similar to H-ras, the total amount of K-ras bound to PM increased after TGFβ-1 addition (Fig. 1c, d, S2).

To study the mechanism of increased membrane localization of H-ras in more detail we tested its truncated version without the catalytic domain (G-domain). It has been documented that the minimal membrane anchor part of H-ras (tH) requires presence of adjacent hypervariable linker region to be laterally segregated as H-ras G12V and K-ras G12V into cholesterol-independent microdomains where the signaling occurs^17,19^. Therefore, we used CTH construct composed of both membrane anchor part and the hypervariable linker region tagged with CFP (Fig. 1a). Indeed, CTH followed the trend set by H-ras indicating that this membrane anchor part is sufficient for the protein response to TGFβ-1 treatment (Fig. 1b–d, S2).

Our results thus show that TGFβ-1 triggers relocalization of H-ras from cytoplasm to PM and causes increased accumulation of K-ras in PM.

### TGFβ-1 treatment triggers rise in positive membrane curvature

In a previous study we have proven on example of tN-ras, a minimal membrane anchor of the N-ras isoform, that Ras senses positive membrane curvature in in vitro reconstituted systems and that this membrane partitioning is essential for its enrichment in raft-like liquid ordered phases of membrane^13^. In addition, H-ras and to a lesser extend also K-ras were shown to preferentially localize to positively curved membranes in vivo^14^. Therefore we decided to investigate if changes in the partitioning of H-ras G12V, CTH and K-ras G12V during TGFβ-1 treatment are accompanied by the acquisition of positive membrane curvature. We transfected MCF7 cells with YFP tagged Nadrin N-BAR, a sensor of positive membrane curvature^20^, and followed changes in Nadrin N-BAR localization triggered by TGFβ-1 (Fig. S1b).

**Figure 2:**
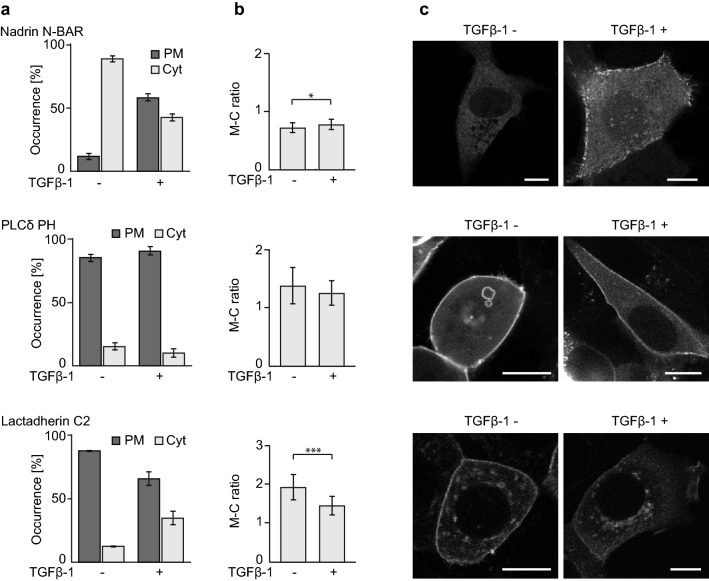
EMT promotes changes in membrane curvature. Comparison of MCF7 cells expressing Nadrin-YFP (Nadrin N-BAR), PLCdelta-GFP (PLCδ PH) and Lact-GFP (Lactadherin C2) domains treated or not with TGFβ-1 for 2 days. Data shown comes from merging two or three independent biological replicates of each protein (for separate analysis of all replicates see Fig. S2). (**a**) Comparison of ratios between cells with cytoplasmic (Cyt) and plasma membrane (PM) localization (Nadrin N-BAR *n* ≥ 93, PLCδ PH *n* ≥ 65, Lactadherin C2 *n* ≥ 168), error bars represent ± 0.5 SD. (**b**) Comparison of membrane-to-cytoplasm ratios (M-C ratio) (Nadrin N-BAR *n* ≥ 132, PLCδ PH *n* ≥ 96, Lactadherin C2 *n* ≥ 188), error bars represent ± 0.5 SD, stars indicate statistically significant differences (*p = 0.01–0.05, **p = 0.001–0.009, ***p < 0.001; for exact *p*-values see Table S2). (**c**) Representative images of all tested conditions. Scale bar 10 μm.

At steady state Nadrin N-BAR localized almost entirely in cytoplasm, whereas several puncta of Nadrin N-BAR accumulated on the PM after treatment with TGFβ-1 indicating that the cells acquired increased positive membrane curvature (Fig. 2).

Apart from sensing positive membrane curvature the N-BAR domains were documented to bind negatively charged lipids. N-BAR domain of another N-BAR containing protein amphiphysin was shown to interact equally well with two lipids—PI(4,5)P2 and phosphatidylserine (PS)^21^. To investigate the possible role of these protein-lipid interactions in observed responses of Nadrin N-BAR to TGFβ-1 we transfected MCF7 cells with GFP-tagged PH domain of PLCdelta, sensor of PI(4,5)P2^22^, and C2 domain of Lactadherin, specifically binding PS^23^(Fig. S1b).

In both cases the sensors of negatively charged lipids showed mostly PM localization that either remained unchanged (PLCdelta PH) or decreased (Lactadherin C2) after TGFβ-1 treatment. Similarly, the total amount of proteins on PM after TGFβ-1 treatment showed no significant difference in case of PLCdelta PH, but decreased significantly in case of Lactadherin C2 (Fig. 2). The effect observed for Lactadherin C2 is likely to be caused by redistribution of PS from PM because a proteomic study performed in MDCK cells did not detect any significant drop in total PS amount after EMT induction^24^. It is also consistent with observation that PM localization of PS decreases with increasing positive membrane curvature whereas localization of PI(4,5)P2 is not significantly affected^14^. Since the response of Nadrin N-BAR to TGFβ-1 treatment does not follow the trend observed in either PLCdelta PH or Lactadherin C2, we can therefore conclude that TGFβ-1 induced changes in Nadrin N-BAR PM localization are solely driven by changes in membrane curvature and indicate rise in positive membrane curvature.

### Increased level of positive membrane curvature leads to elevated H-ras PM localization

To validate our theory that the increase in accumulation of Ras proteins in PM during TGFβ-1 treatment is driven by the rise of positive membrane curvature we performed a set of further experiments for which we used a fibroblast cell line NIH 3T3 as an independent system. NIH 3T3 cells were selected because fibroblasts are rich in caveolae, small PM invaginations about 60 nm in size, and thus contain high number of areas with positive membrane curvature^25^. Indeed, both the M-C ratio and the proportion of cells with PM localization of Nadrin N-BAR were higher in NIH 3T3 cells than in MCF7 cells before or after TGFβ-1 treatment (Fig. S3). Similarly, both H-ras and CTH showed significantly higher association with PM in NIH 3T3 compared to situation in MCF 7 favoring the fact that H-ras and CTH also recognize positive membrane curvature (Fig. S3).

In contrast, recruitment of K-ras to PM decreased in NIH 3T3 compared to MCF7 cells (Fig. S3c). This result is consistent with previous observation in BHK cells where increase in positive membrane curvature also led to PM depletion of K-ras^14^. A possible explanation for this could lay in a different way how H-ras and K-ras associate with PM. PM localization of H-ras occurs through two lipid anchors—farnesyl and palmitoyl, whereas PM localization of K-ras, apart from its one lipid anchor (C-terminal farnesyl), largely depends on binding negatively charged lipids, especially phosphatidylserine (PS), via its polybasic domain (PBD)^26,27^. Similar to K-ras, Lactadherin C2 showed also significantly reduced PM binding (Fig. S3e). Moreover, it was recently demonstrated that K-ras G12V strictly prefers PS with unsaturated acyl chains over fully saturated PS^28^. Since caveolae are known to be composed mostly of lipids with saturated acyl chains, pool of unsaturated PS available for binding may be actually significantly smaller in caveolae-rich fibroblasts compared to epithelia cells and, in combination with overall lower PS level, it could represent a main limiting factor for K-ras G12V PM recruitment in NIH 3T3 cells.

**Figure 3:**
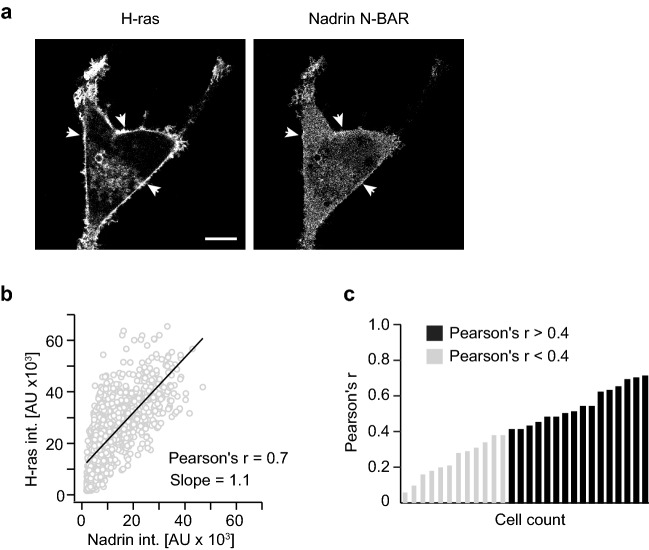
H-ras colocalize on PM with Nadrin N-BAR domain. (**a**) Illustrative NIH 3T3 cell co-expressing GFP-H-ras G12V (H-ras) and Nadrin-YFP (Nadrin N-BAR), arrows point to areas of collocalization of signals. Scale bar 10 μm. (**b**) Plot showing correlation of H-ras and Nadrin N-BAR fluorescence signals from PM of the cell shown in a). Pearson’s r = 0.7, slope = 1.1. (**c**) Plot summarizing Pearson’s r calculated from correlation of fluorescence signals from co-expressed H-ras and Nadrin N-BAR (*n* = 28). Pearson’s r > 0.4 represents a threshold for moderate-to-strong correlation between two variables.

To inspect if Ras proteins indeed preferentially localize to areas of high positive membrane curvature we co-transfected H-ras G12V with Nadrin N-BAR in NIH 3T3 cells and we observed a significant correlation in PM localization of both proteins (Fig. 3).

### Disruption of membrane curvature causes drop in Ras proteins PM localization

The results of our experiments strongly suggests that recognition of positive membrane curvature is likely a driving mechanism behind increased PM targeting of Ras after TGFβ-1 treatment. Therefore, experiments reducing positive membrane curvature of cell membranes should cause release of Ras from PM.

**Figure 4:**
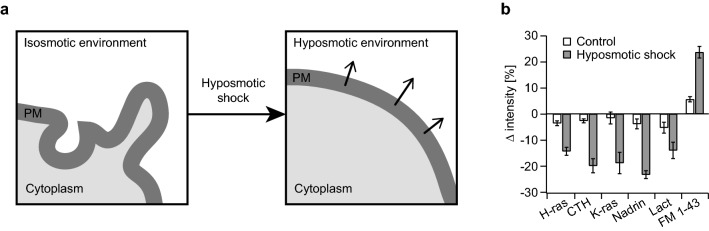
H-ras, CTH, and K-ras are released from PM after hyposmotic shock. NIH 3T3 expressing GFP-H-ras G12V (H-ras), CFP-CTH (CTH), GFP-K-ras G12V (K-ras), Lact-GFP (Lact) and Nadrin-YFP (Nadrin N-BAR), or stained with plasma membrane dye FM1-43 were subjected to hyposmotic shock. (**a**) A scheme illustrating impact of hyposmotic shock on PM membrane curvature. (**b**) Plot summarizes changes in normalized integrated membrane intensity of fusion proteins caused by hypoosmotic shock. The difference is calculated as % change in intensity between a mean value of 3 images taken within the first 60 s of imaging (i.e. before hyposmotic shock) and mean value of 4 images taken at the end of imaging.

One option how to reduce membrane curvature is to subject cells to hyposmotic shock. The cells start to swell as indicated by increased FM1-43 staining (Figs. 4a, b, S4)^29^. Moreover, osmotic swelling leads to rapid disappearance of caveolae which is more prominent the lower the osmolarity gets. For our experiments we thus used hyposmotic level shown to be required to reduce caveolae by ~ 30%^30^. Drop in Nadrin N-BAR PM localization indeed confirmed that hyposmotic shock reduces positive membrane curvature in NIH 3T3 cells. H-ras and CTH were both rapidly released from PM following hyposmotic shock induction with response of CTH being more pronounced than the one of H-ras. K-ras also showed fast and stable release from plasma membrane (Figs. 4b, S4). Interestingly, the hyposmotic shock decreased the level of Lactadherin C2 bound to PM as well which is in contradiction with observation that PM localization of PS decreases with increasing positive membrane curvature (Fig. 2, and^14^). One possible explanation could be that PS in PM respond differently to changing membrane curvature under different conditions. Alternative explanation could be that, in addition to stretching the membrane and disappearance of caveolae, the high hyposmotic stress may disrupt the global organization of the membrane bilayer which may then contribute to the loss of interactions of some proteins with PM^14^. Both of these possibilities could provide an explanation to the release of Lactadherin C2 from PM following hyposmotic shock and may contribute also to the release of K-ras taking into account the dependence on PS binding of both of these proteins (Figs. 4b, S4). The drop in K-ras PM localization was much higher than in case of Lactadherin C2 suggesting that reduction in PM PS level is likely not the only reason for K-ras release from PM and that reduction in positive membrane curvature also plays its role. However, the different level of response of K-ras and Lactadherin C2 to the hyposmotic shock may have one more explanation. It was previously shown that distinct PS species prefer membranes of different curvature depending on their acyl chains—fully saturated and mono-unsaturated PS species favor highly curved membranes, while the mixed-chain PS species prefer less curved membranes^14^. The distinct PS pools in PM may respond to changes in membrane curvature in opposing ways, yielding a more subtle response of the global PS localization on the PM (sensed by Lactadherin C2) in comparison with K-ras G12V which was shown to prefer PS with unsaturated acyl chains over fully saturated PS^28^.

In contrast to Nadrin N-BAR that showed constant and gradual decrease in PM localization, the release of Ras isoforms from plasma membrane reaches sooner or later plateau reflecting possibly different ways of interaction with PM and membrane curvature recognition by these proteins (concave shape of N-BAR vs. lipid anchors of Ras) (Fig. S4).

**Figure 5:**
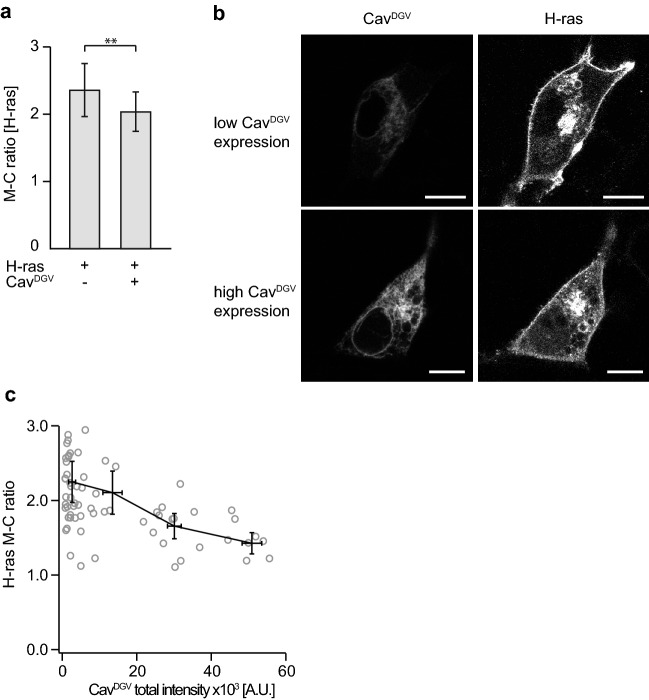
Increasing CavDGV expression causes relocalization of H-ras from PM to cytoplasm. (**a**) Plot shows comparison of M-C ratio of RFP-H-ras G12V (H-ras) in NIH 3T3 cells co-expressing RFP-H-ras G12V (H-ras) and GFP-CavDGV (CavDGV) (*n* = 73), or expressing RFP-H-ras G12V (H-ras) alone (*n* = 65). Error bars represent ± 0.5 SD. Stars indicate statistically significant differences (**p = 0.001–0.009; for exact p-values see Table S3). (**b**) Impact of GFP-CavDGV (CavDGV) expression level on subcellular localization of RFP-H-ras G12V (H-ras). Scale bar 10 μm. (**c**) Plot shows dependence of RFP-H-ras G12V (H-ras) M-C ratio on expression levels of GFP-CavDGV (CavDGV) from co-expression experiment in NIH 3T3 cells. M-C ratio was calculated as described in the materials and methods section. The expression levels of CavDGV was calculated by summing up the integrated intensity of the membrane and cytosol ROIs. Data was divided into four bins according to the expression level (0–8000, 8000–25,000, 25,000–40,000, 40,000–65,000 a.u.) and average M-C ratio with average expression level was represented by the black crosses. Error bars of these crosses represent the SEM.

Besides the hyposmotic shock that disturbs membrane curvature in general, positive membrane curvature can be specifically reduced by targeted lowering of the caveolae number. Expression of dominant negative caveolin (Cav^DGV^) was shown to significantly reduce number of caveolae (reduction by 62% in BHK cells)^31^. We performed co-expression of GFP-Cav^DGV^ with RFP-H-ras G12V in NIH 3T3 cells followed by quantitative analysis of H-ras PM localization. Indeed, we observed a decrease in H-ras PM localization when single (RFP-H-ras G12V) and double (GFP-Cav^DGV^ with RFP-H-ras G12V) transfected cells were compared (Figs. 5, S5). The reduction was partial because, consistently with previous observations, H-ras was detectable on PM even at high expression levels of Cav^DGV^ (Figs. 5, S5);^31^. However, the effect of Cav^DGV^ expression on the PM localization of H-ras was even more evident from the negative correlation between the amount of H-ras associated with PM and the level of Cav^DGV^ expression (Figs. 5, S5).

## Discussion

Ras proteins are small GTPases that serve as important regulators of cell pathways responsible for proliferation, differentiation and cell survival. Mutations in key conserved sites within Ras proteins lead to elevated GTP binding which results in constitutive activation of Ras. Among the three Ras isoforms, mutations in K-ras are the most frequently detected in human cancer, but specific associations of individual mutated Ras isoforms with particular cancer types were detected^3,4^.

Similarly as Ras proteins, TGFβ-1 is frequently overexpressed in human tumours^32^ and its expression is generally associated with poor prognosis^33,34^. One of the functions of TGFβ-1 is to induce EMT, which is important biological process critical during embryogenesis, but it is also exploited by cancer cells during tumor progression. TGFβ-1 signal alone was shown to be insufficient for acquisition of invasive potential of cancerous cells. To gain full invasive potential activated Ras (H-ras) that alters TGFβ-1 response is needed^10^. Moreover, activated Ras (H-ras) or its downstream effectors are important for promoting EMT through autocrine production of TGFβ-1 and continuous TGFβ-1 signaling^8,9^.

For proper Ras signaling its PM localization is essential. In this study we showed that TGFβ-1 treatment of breast cancer cells MCF7 is followed by elevated PM localization of activated H-ras and K-ras. Suggesting that TGFβ-1 itself can promote Ras PM residence. The driving mechanism behind this seemed to be triggered by TGFβ-1 and manifested by alterations in membrane curvature resulting in increased level of positive membrane curvature. The recognition of membrane curvature by activated Ras is likely important for proper signaling, because it is known that Ras signaling activity is localized mostly at the periphery of a cell or the leading edge of a migrating cell, where membrane ruffling is prominent in both cases^35,36^.

Our results provide evidences supporting hypothesis that Ras proteins are indeed able to react to changes in membrane curvature in vivo which is in agreement with recent observations by others^14^. The membrane anchor part of H-ras seems to be central and sufficient to H-ras recognition of positive membrane curvature. H-ras, containing two lipid anchors, seems to be also more potent sensor of positive membrane curvature than K-ras that contains only one lipid anchor, which is consistent with our previous finding that the presence of more lipid anchors leads to higher sensitivity to positive membrane curvature^12^. Active K-ras indeed prefers less curved membranes than active H-ras even in vivo and increase in positive curvature rather leads to its disappearance from PM^14^. Despite the fact that PM localization of K-ras largely depends on the presence of PS, its membrane partitioning driven by TGFβ-1 treatment seems to be at least partially PS independent as the amount of K-ras on PM increases during TGFβ-1 treatment, whereas amount of specific PS sensor Lactadherin C2 decreases (Figs. 1 and 2, S2).

**Figure 6:**
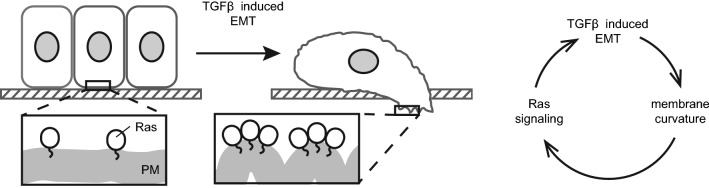
Model of the MC dependent regulation of Ras during TGFβ-1 induced EMT.

The effect of TGFβ-1 treatment on Ras PM localization described in this paper suggests a simple mechanism of possible positive feedback loop within previously described TGFβ-1 and Ras cooperation during cancer progression. In this hypothesis an increased level of membrane curvature of PM during TGFβ-1 induced EMT would attract more Ras molecules to PM resulting in its facilitated activation and subsequent further promotion of EMT and acquisition of invasive potential (Fig. 6). However, further validation of this theory would be needed. Especially a detailed insight into the molecular mechanism behind observed phenotypes would bring broader understanding of the suggested link between membrane curvature sensing of Ras proteins and TGFβ-1 induced EMT and could be achieved for example via studying the process after inhibition of TGFβ-1 induced signaling pathways^37,38^. It is noteworthy that our experiments with CTH suggest that the hyperactivating G12V mutation is not necessary for curvature coupling of H-Ras. The novel TGFβ-EMT feedback loop we propose here, which leads to H-Ras membrane localization and activation, is mediated by the membrane curvature biophysical properties of the CTH anchor. The mechanism of membrane-curvature-based H-Ras activation is thus, in principle, not predicated on the existence of mutations. However, this mechanism will be particularly sensitive to stimuli that modify plasma membrane morphology during both development and tumor formation^39,40^. Last but not least, it is important to highlight that the pathological relevance of the observed effect of TGFβ-1 treatment on PM localization of different Ras isoforms for cancer progression is currently unclear and should be the subject of further studies.

## Methods

### Cell lines and plasmids

MCF 7 cells, provided by Prof. Moshe Oren (Weizmann Institute of Science), and NIH 3T3 cells (ATCC CRL-1658) were maintained in culturing medium composed of DMEM medium (GIBCO, Thermo Fisher Scientific) supplemented with 10% FBS (GIBCO, Thermo Fisher Scientific) at 37C and 5% CO_2_.

The CFP-CTH (lipid anchor of H-ras, AA 166-189)^19^ and GFP-H-ras G12V were supplied by Prof. Daniel Abankwa (University of Luxemburg)^17^, the GFP-K-ras G12V were kind gift of Professor John F. Hancock (University of Texas)^41^, RFP-H-ras G12V and GFP-Cav^DGV^ plasmid were provided by Professor Robert G. Parton (Institute of Molecular Bioscience, University of Queensland)^31^. The Nadrin-YFP plasmid (YFP fusion of a BAR domain of nadrin 2 protein, AA 1-244 + 10 AA linker) was provided by Dr. Milos Galic (Insitute of Medical Physics and Biophysics, University of Münster)^20^. PLCδ-GFP (GFP fusion of PH-domain of rat PLCδ protein), and Lact-GFP (GFP fusion of C2-domain of bovine protein Lactadherin) plasmids were a kind gift from Dr. Carsten Schultz (EMBL Heidelberg, Germany).

### Plating of cells and transfection

Cells were grown in 6 well plates on round cover slips (Ø 18 mm, VWR) that were thoroughly cleaned using 2% Hellmanex III (Hellma^®^Analytics) and MetOH prior use. In case of MCF7 cells 6 × 10^4^ of cells were plated per well, in case of NIH 3T3 5 × 10^4^ of cells were plated per well. For co-transfection experiment of GFP-Cav^DGV^ with RFP-H-ras G12V 10 × 10^4^ NIH 3T3 cells were plated per well. Cells were grown for 24 h in culturing medium before transfection. For co-transfection experiment of RFP-H-ras G12V and Nadrin-YFP N-BAR plasmids 5 × 10^4^ of cells were plated and 1 μg of each plasmid was used. The transfection was performed using Lipofectamine plus (Invitrogin) according to manufacturer’s instructions.

### TGFβ-1 treatment

24 h after transfection the transfected cells were placed in fresh culturing medium supplemented with 2 ng/ml of TGFβ-1(R&D systems) and incubated in this medium for two days.

### Live cell imaging

For imaging the cover slips were mounted in custom made microscopy chambers with total volume of 90 μL. Cells were imaged in imaging medium (DMEM without phenol red with 10% FBS) using Leica TCS SP5 inverted confocal microscope with a water immersion objective HCX PL APO CS × 63 (NA 1.2). Signal from GFP was detected at 495–590 nm (exc. 488 nm) and YFP was detected at 520–538 nm (exc. 514 nm); CFP was detected at 468–590 nm (exc. 458 nm); RFP was detected at 565–699 nm (exc. 543 nm); FM 143 was detected at 560–610 nm (exc. 471 nm). In case of co-expression of proteins with two different fluorescent tags sequential imaging was used to avoid cross excitation. Images had a resolution of 2048 × 2048 pixels, with a pixel size of 120 nm and a 16-bit depth. All acquired images were then processed with open source software Fiji.

### Hyposmotic shock

We imposed hyposmotic shock following the protocol described in^20^. Cover slips with transfected NIH 3T3 cells were mounted into microscopy chambers and imaged (sequential imaging, one image taken every 30 s). After one minute of imaging the full chamber volume (90 μL) of imaging medium was replaced with imaging medium diluted with milliQ water in ratio 1:6 (from 300 to 50 mM DMEM) and the sample was imaged further for another 5 min. The control sample was treated in the same way except that the imaging medium was replaced with 90 uL of fresh non-diluted imaging medium. Membrane swelling after application of diluted medium was checked visually and also by plasma membrane staining with FM 143 dye (Thermo Fisher Scientific). The staining was performed according to manufacturer’s instructions.

### Quantitative image analysis

Quantitative image analysis procedures were performed in IgorPro (version 6.37, Wavemetrics, USA). Only healthy cells in focus and with clear borders (identified by eye) were used for the analysis. First to visualize cell membrane better and to reduce noise images were convoluted with a Gaussian blur filter (5 pixels size). Then line ROI (5 pixels width) was drawn manually on top of the plasma membrane. This line ROI allowed us to isolate pixels in image belonging to the plasma membrane and cytoplasm. The line ROIs were always drawn as close to cell interior as possible. In this way we minimized the potential impact of the fluorescent signal coming from the adjacent cell(s) on plasma membrane intensity value of the cell of interest in cases when two or more cells were in close contact. Finally, based on the line ROI, statistics of fluorescence intensity in the plasma membrane and cytoplasm pixels were calculated using the raw image. The membrane and cytoplasm intensity ratio (M-C ratio) was assessed by dividing average membrane intensity by average cytoplasm intensity. To find whether the difference of M-C ratio between two samples is statistically significant, we performed two-tailed T-test (significance level = 0.05, equivalent degrees of freedom accounting for possibly different variances).

For analysis of data from hypoosmotic shock experiment integrated intensity of the membrane was used for making intensity versus time traces. In order to be able to compare the osmotic shock effect between different samples each trace was normalized to the average intensity of the first three points from the beginning of the trace. Next an average intensity for first 3 points (I0) and from point 4 till the end of the trace (I1) was calculated and used for the percentage intensity change ($\Delta I$) calculation. Then $\Delta I = \left( {\frac{I1 - I0}{{I0}}} \right)*100$ was calculated for each individual cell and their average and SD was taken.

To analyse co-expression experiment of GFP-Cav^DGV^ with RFP-H-ras G12V in NIH 3T3 cells the linear correlation test was performed and the linear correlation coefficient and its standard error were calulated to estimate the degree of correlation between H-ras M-C ratio and the total Cav^DGV^ expression. The significance level was set to 0.05, that gave confidence intervals for the correlation coefficient at 95%.

### Qualitative image analysis

Qualitative image analysis was based on visual inspection of acquired images. According to localization of fluorescent signal coming from expressed fusion proteins the cells were manually classified as showing cytoplasm localization (i.e. fluorescent signal was visible only in cytoplasm with no plasma membrane localization) or plasma membrane localization (i.e. fluorescent signal was localized entirely or at least partially on plasma membrane) of expressed protein. Only healthy cells in focus and with clear borders were assessed in the analysis.

## Supplementary Information

Supplementary Information 1.

Supplementary Information 2.

## Data Availability

All data are contained within the manuscript.

## References

[1] Simanshu DK, Nissley DV, McCormick F (2017). RAS proteins and their regulators in human disease. Cell.

[2] Cox AD, Der CJ (2010). Ras history: the saga continues. Small GTPases.

[3] Prior IA, Lewis PD, Mattos C (2012). A comprehensive survey of Ras mutations in cancer. Cancer Res..

[4] Cox AD, Fesik SW, Kimmelman AC, Luo J, Der CJ (2014). Drugging the undruggable RAS: mission possible?. Nat. Rev. Drug Discov..

[5] Zavadil J, Bottinger EP (2005). TGF-beta and epithelial-to-mesenchymal transitions. Oncogene.

[6] Schneider D (2013). Tension monitoring during epithelial-to-mesenchymal transition links the switch of phenotype to expression of moesin and cadherins in NMuMG cells. PLoS ONE.

[7] Zhang YE (2017). Non-Smad Signaling Pathways of the TGF-beta Family. Cold Spring Harb. Perspect. Biol..

[8] Oft M (1996). TGF-beta1 and Ha-Ras collaborate in modulating the phenotypic plasticity and invasiveness of epithelial tumor cells. Genes Dev..

[9] Lehmann K (2000). Raf induces TGFbeta production while blocking its apoptotic but not invasive responses: a mechanism leading to increased malignancy in epithelial cells. Genes Dev..

[10] Safina AF (2009). Ras alters epithelial-mesenchymal transition in response to TGFbeta by reducing actin fibers and cell-matrix adhesion. Cell Cycle.

[11] Grusch M (2010). The crosstalk of RAS with the TGF-beta family during carcinoma progression and its implications for targeted cancer therapy. Curr. Cancer Drug Targets.

[12] Hatzakis NS (2009). How curved membranes recruit amphipathic helices and protein anchoring motifs. Nat. Chem. Biol..

[13] Larsen JB (2015). Membrane curvature enables N-Ras lipid anchor sorting to liquid-ordered membrane phases. Nat. Chem. Biol..

[14] Liang H (2019). Membrane curvature sensing of the lipid-anchored K-Ras small GTPase. Life Sci. Alliance.

[15] Newlaczyl AU, Coulson JM, Prior IA (2017). Quantification of spatiotemporal patterns of Ras isoform expression during development. Sci. Rep..

[16] Mageean CJ, Griffiths JR, Smith DL, Clague MJ, Prior IA (2015). Absolute quantification of endogenous Ras isoform abundance. PLoS ONE.

[17] Prior IA, Muncke C, Parton RG, Hancock JF (2003). Direct visualization of Ras proteins in spatially distinct cell surface microdomains. J. Cell Biol..

[18] Hancock JF (2003). Ras proteins: different signals from different locations. Nat. Rev. Mol. Cell Biol..

[19] Rotblat B (2004). Three separable domains regulate GTP-dependent association of H-ras with the plasma membrane. Mol. Cell Biol..

[20] Galic M (2012). External push and internal pull forces recruit curvature-sensing N-BAR domain proteins to the plasma membrane. Nat. Cell Biol..

[21] Saarikangas J (2009). Molecular mechanisms of membrane deformation by I-BAR domain proteins. Curr. Biol..

[22] Lemmon MA, Ferguson KM, O'Brien R, Sigler PB, Schlessinger J (1995). Specific and high-affinity binding of inositol phosphates to an isolated pleckstrin homology domain. Proc. Natl. Acad. Sci. U. S. A..

[23] Yeung T (2008). Membrane phosphatidylserine regulates surface charge and protein localization. Science.

[24] Sampaio JL (2011). Membrane lipidome of an epithelial cell line. Proc. Natl. Acad. Sci. U. S. A..

[25] Sotgia F (2012). Caveolin-1 and cancer metabolism in the tumor microenvironment: markers, models, and mechanisms. Annu. Rev. Pathol..

[26] Apolloni A, Prior IA, Lindsay M, Parton RG, Hancock JF (2000). H-ras but not K-ras traffics to the plasma membrane through the exocytic pathway. Mol. Cell Biol..

[27] Cho KJ (2016). Inhibition of acid sphingomyelinase depletes cellular phosphatidylserine and mislocalizes K-Ras from the plasma membrane. Mol. Cell Biol..

[28] Zhou Y (2017). Lipid-sorting specificity encoded in k-ras membrane anchor regulates signal output. Cell.

[29] Masters TA, Pontes B, Viasnoff V, Li Y, Gauthier NC (2013). Plasma membrane tension orchestrates membrane trafficking, cytoskeletal remodeling, and biochemical signaling during phagocytosis. Proc. Natl. Acad. Sci. U. S. A..

[30] Sinha B (2011). Cells respond to mechanical stress by rapid disassembly of caveolae. Cell.

[31] Roy S (1999). Dominant-negative caveolin inhibits H-Ras function by disrupting cholesterol-rich plasma membrane domains. Nat. Cell Biol..

[32] Derynck R (1985). Human transforming growth factor-beta complementary DNA sequence and expression in normal and transformed cells. Nature.

[33] Friedman E (1995). High levels of transforming growth factor beta 1 correlate with disease progression in human colon cancer. Cancer Epidemiol. Biomarkers Prev..

[34] Shim KS, Kim KH, Han WS, Park EB (1999). Elevated serum levels of transforming growth factor-beta1 in patients with colorectal carcinoma: its association with tumor progression and its significant decrease after curative surgical resection. Cancer.

[35] Mochizuki N (2001). Spatio-temporal images of growth-factor-induced activation of Ras and Rap1. Nature.

[36] Sasaki AT, Chun C, Takeda K, Firtel RA (2004). Localized Ras signaling at the leading edge regulates PI3K, cell polarity, and directional cell movement. J. Cell Biol..

[37] Lamouille S, Derynck R (2007). Cell size and invasion in TGF-beta-induced epithelial to mesenchymal transition is regulated by activation of the mTOR pathway. J. Cell Biol..

[38] Eger A (2004). beta-Catenin and TGFbeta signalling cooperate to maintain a mesenchymal phenotype after FosER-induced epithelial to mesenchymal transition. Oncogene.

[39] Engler AJ, Humbert PO, Wehrle-Haller B, Weaver VM (2009). Multiscale modeling of form and function. Science.

[40] Hayward MK, Muncie JM, Weaver VM (2021). Tissue mechanics in stem cell fate, development, and cancer. Dev. Cell.

[41] Prior IA (2001). GTP-dependent segregation of H-ras from lipid rafts is required for biological activity. Nat. Cell Biol..

