# Supplementary material for: TGFβ-induced changes in membrane curvature influence Ras oncoprotein membrane localization
Source: Sci Rep. 2022 Aug 5;12:13486. doi: 10.1038/s41598-022-17482-8 (PMC9356053; doi:10.1038/s41598-022-17482-8)
Supplement: Supplementary file 1 — Supplementary Information 1. [file 41598_2022_17482_MOESM1_ESM.pdf]

TGFβ-induced changes in membrane curvature influence Ras oncoprotein membrane localization

Alexandros Damalas, Ivana Vonkova, Marijonas Tutkus, Dimitrios Stamou

Department of Chemistry, University of Copenhagen,
Copenhagen, Denmark.

Supplementary information

Content

Figure S1...S-2

Figure S2...S-3

Figure S3...S-4

Figure S4...S-5

Figure S5...S-6

Supplementary Tables S1-S5

Figure S1.

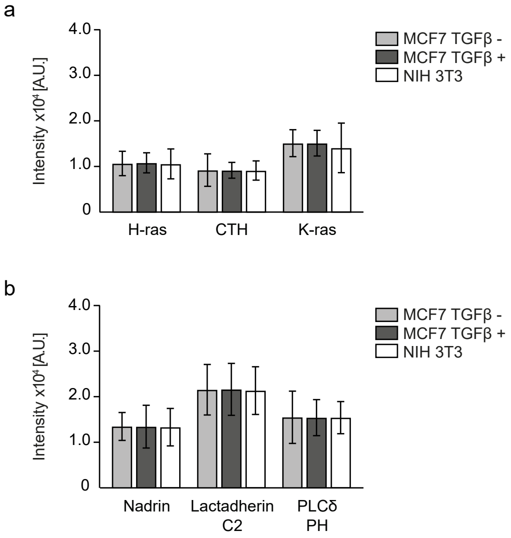

Figure S1. Comparison of expression levels
Comparisons of the total expression levels of a) GFP-H-ras G12V (H-ras; MCF7 n≥94, NIH 3T3 n=45), CFP-CTH (CTH; MCF7 n≥80, NIH 3T3 n=65), GFP-K-ras G12V (K-ras; MCF7 n≥199, NIH 3T3 n=24); and b) Nadrin-YFP (Nadrin N-BAR; MCF7 n≥132, NIH 3T3 n=52), Lact-GFP (Lactadherin C2; MCF7 n≥188, NIH 3T3 n=72), and PLCδ-GFP (PLCδ PH; MCF7 n≥96, NIH 3T3 n=42) expressed in MCF7 and treated or not for 2 days with TGFβ-1 or expressed in NIH 3T3 cells.
Error bars represent ±0.5 SD.

Figure S2.

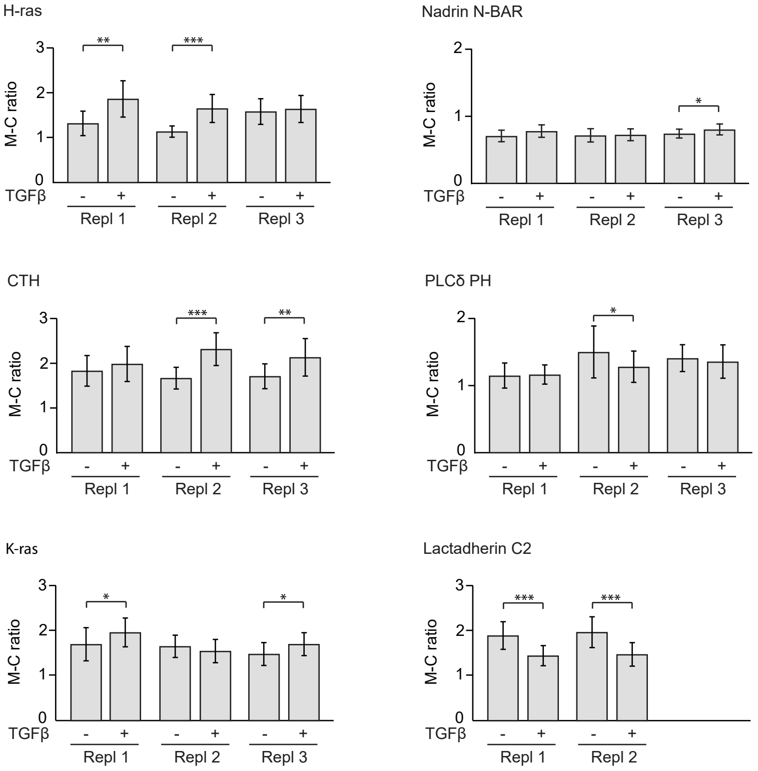

Figure S2. Impact of TGFβ-1 on PM localization of Ras proteins and controls
Comparison of M-C ratio of individual replicates of MCF7 cells expressing GFP-H-ras G12V (H-ras, n≥18), CFP-CTH (CTH, n≥15), GFP-K-ras G12V (K-ras, n≥46) and Nadrin-YFP (Nadrin N-BAR, n≥36), PLCdelta-GFP (PLCδ PH, n≥26), and Lact-GFP (Lactadherin C2, n≥72) treated or not with TGFβ-1 for 2 days. In all cases error bars represent ±0.5 SD, stars indicate statistically significant differences (* p = 0.01-0.05, ** p = 0.001-0.009, *** p < 0.001; for exact p-values see Table S4). Repl, replicate.

Figure S3.

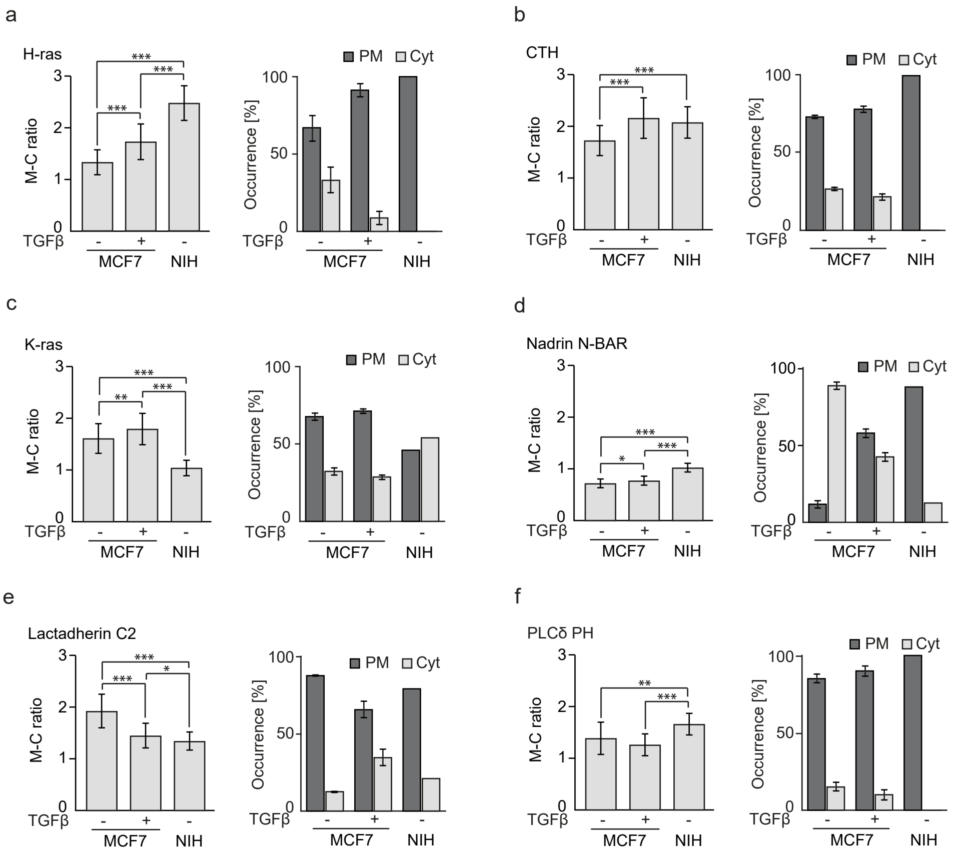

Figure S3. Comparison of subcellular localization of Ras proteins and controls in MCF7 and NIH3T3 cells

Comparison of proteins expressed in MCF7 cells treated or not with TGFβ-1 for 2 days and in steady state NIH 3T3 cells. Plots in the left show comparison of M-C ratio; plots in the right compare ratios between cells with cytoplasmic (Cyt) and plasma membrane (PM) localization. Error bars represent ±0.5 SD, stars indicate statistically significant differences (* p = 0.01-0.05, ** p = 0.001-0.009, *** p < 0.001; for exact p-values see Table S5).

a) GFP-H-ras G12V (H-ras), b) CFP-CTH (CTH), c) GFP-K-ras G12V (K-ras), d) Nadrin-YFP (Nadrin N-BAR), e) Lact-GFP (Lactadherin C2), f) PLCdelta-GFP (PLCδ PH).

Figure S4.

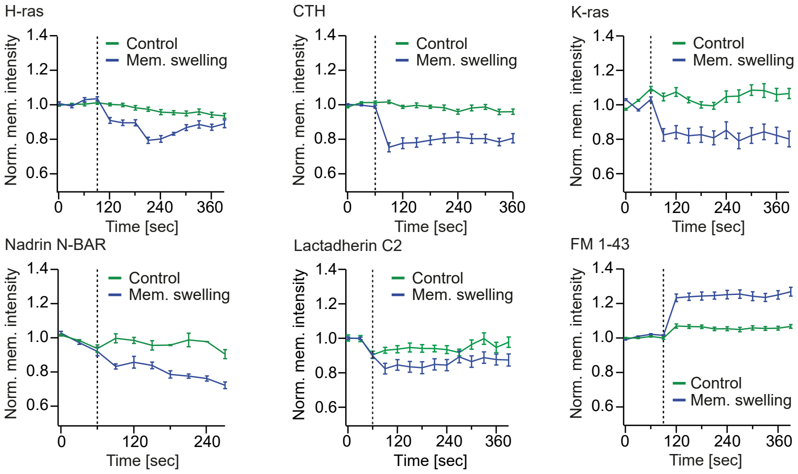

Figure S4. H-ras, CTH, and K-ras are released from the membrane after hyposmotic shock. NIH 3T3 expressing GFP-H-ras G12V (H-ras), CFP-CTH (CTH), GFP-K-ras G12V (K-ras), Lact-GFP (Lact) and Nadrin-YFP (Nadrin NBAR), or stained with plasma membrane dye FM1-43 were subjected to hyposmotic shock. Plots show timecourse of hyposmotic shock on normalized integrated membrane intensity of fusion proteins/dye. Error bars represent ±0.5 SD. Time of addition of hypotonic medium is indicated by a dashed line.

Figure S5.

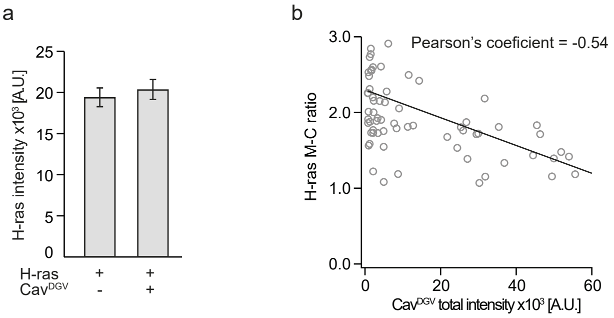

Figure S5. Increasing Cav^{DGV} expression causes relocalization of H-ras from PM to cytoplasm
a) Comparison of the total expression levels of RFP-H-ras G12V (H-ras) in cells co-expressing RFP-H-ras G12V (H-ras) and GFP-Cav^{DGV} (Cav^{DGV})(n = 73) or expressing RFP-H-ras G12V (H-ras) alone (n=65).
b) Plot shows dependence of RFP-H-ras G12V (H-ras) M-C ratio on expression levels of GFP-Cav^{DGV} (Cav^{DGV}) from co-expression experiment in NIH 3T3 cells (n = 73). The negative correlation of the two values is represented by Pearson's coefficient of -0.54.
